# The time course of nod-like receptor family pyrin domain containing 3 inflammasome complex expressions in the testis tissue of an experimental varicocele rat model: An experimental study

**DOI:** 10.18502/ijrm.v21i7.13895

**Published:** 2023-08-23

**Authors:** Hamideh Fallah Asl, Farideh Jalali Mashayekhi, Adib Zendedel, Maryam Baazm

**Affiliations:** ^1^Students Research Committee, Arak University of Medical Sciences, Arak, Iran.; ^2^Department of Genetics and Biochemistry, School of Medicine, Arak University of Medical Sciences, Arak, Iran.; ^3^Institute of Neuroanatomy, Uniklinik RWTH Aachen, 52074 Aachen, Germany.; ^4^Department of Anatomy, School of Medicine, Arak University of Medical Sciences, Arak, Iran.; ^5^Molecular and Medicine Research Center, Arak University of Medical Sciences, Arak, Iran.

**Keywords:** Varicocele, Inflammasomes, NLRP3, Rat.

## Abstract

**Background:**

Varicocele is characterized by abnormal dilation of the testicular vein, which results in hypoxia, the accumulation of reactive oxygen species, and the production of proinflammatory cytokines. It seems that a group of cytosolic receptors named nod-like receptor family, pyrin domain containing 3 (NLRP3) inflammasome, is activated and involved in the pathogenesis of varicocele.

**Objective:**

We aim to determine the time course of NLRP3 inflammasome expression in the testis tissue following varicocele induction.

**Materials and Methods:**

In this experimental study, 36 adult Wistar rats (8 wk, 200-250 gr) were used. For the varicocele induction, the left renal vein was partially ligated. The mRNA levels of NLRP3, apoptosis-associated speck-like protein containing a caspase recruitment domain, and caspase-1 were evaluated by real-time polymerase chain reaction at 1, 2, 4, 8, and 12 wk after varicocele induction.

**Results:**

Results showed that the gene expression of NLRP3 inflammasome component including NLRP3, apoptosis-associated speck-like protein containing a caspase recruitment domain, and caspase-1 did not alter during week 1, 2, 4, and 8 after operation (p = 0.09). 12 wk after varicocele induction, gene expression levels were significantly up-regulated (p = 0.02).

**Conclusion:**

Our data provides clear evidence that varicocele stimulates inflammasome activation in the testis tissue 12 wk after the operation, and this time is required for investigating NLRP3 activity in the varicocele rat model.

## 1. Introduction

Varicocele is one of the male fertility problems caused by abnormal dilation of the testicular veins of the pampiniform plexus (1). This pathological condition impairs testicular tissue, the spermatogenesis process, and sperm function (2). The pathogenesis of varicocele is multifactorial, and several mechanisms are responsible for decreasing fertility in varicocele patients (2, 3).

Varicocele causes an increase in testicular temperature, hypoxia, and overproduction of reactive oxygen species (3, 4), resulting in testicular cell damage and inflammatory responses (5, 6). It has been demonstrated that the semen of varicocele patients contains higher levels of several inflammatory cytokines, such as interleukin (IL)-6, IL-1b, and tumor necrosis factor-alpha (7-9). In addition, the induction of varicocele in immature rats could elevate the expression of IL-6 and interferon-gamma in serum and testis tissue (10).

In response to pathological conditions, such as cell damage, cell stress, and inflammation, the immune system activates a group of cytosolic receptors named inflammasomes (5, 6). The inflammasome family has 4 different members: nod-like receptor (NLR) family pyrin domain containing 1, NLR family pyrin domain containing 3 (NLRP3), NLR family caspase recruitment domain containing 4, and absent in melanoma 2 (5, 6). NLRP3 is a well-known member of the NLR family. NLRP3 and an apoptosis-associated speck-like protein containing an NLR family caspase recruitment domain and caspase-1 form an inflammasome complex, activated by endogenous and exogenous stimulations (6). NLRP3 is an important regulator for caspase-1 that sequentially regulates the transformation of immature proinflammatory cytokines such as IL-1b and IL-18. Upregulation of NLRP3 has been reported in some diseases, such as ischemia (11), Alzheimer's disease (12), and spinal cord injury (SCI) (13). It is believed that the low quality of semen in patients suffering from SCI is related to NLRP3 inflammasome component activity in the semen of these patients and by blocking this pathway it is possible to improve semen quality in these subjects (14). In our previous work, we detected NLRP3, apoptosis-associated speck-like protein containing a caspase recruitment domain (ASC), and IL-1b in the semen of varicocele patients (7). Furthermore, some antioxidants or anti-inflammatory agents, such as resveratrol (15), selenium, and polydeoxyribonucleotide (16), have been shown to reduce varicocele complications by downregulating the NLRP3 inflammasome, reducing apoptosis, and increasing testosterone levels.

Therefore, it seems that overexpression of NLRP3 might be one of the reasons involved in infertility following varicocele. Because an experimental animal model can mimic some pathological conditions in humans and that these studies are critical for understanding the pathophysiology of some diseases, the current study aimed to determine the time of onset of NLRP3 inflammasome component expression after varicocele induction in rats, which will be useful for future varicocele research.

## 2. Materials and Methods

### Animals and surgery

All experiments were performed on 36 adults Wistar rats (8 wk, 200-250 gr, Pasteur, Iran). The animals were kept in a 12 hr light/dark cycle in controlled conditions with free access to water and food.

The animals were randomly divided into 6 groups of 6 animals each. The control animals remained intact, whereas other animals underwent surgery. For varicocele induction, animals were anesthetized with intraperitoneal injections of 100 mg/kg ketamine and 10 mg/kg xylosine (both from Alfasan, Iran). The abdomen was shaved and cleaned, and the skin and muscles were cut. The left renal and spermatic veins were first isolated from the surrounding tissue, and then a 0.85-mm wire was placed parallel to the left renal vein. To ligate the left renal vein, a 4-0 silk suture was wrapped around the wire and the vein. Finally, the wire was gently removed, and the abdominal muscles and skin were sutured (15). Control and varicocele-induced animals were sacrificed under deep anesthesia by ketamine and xylosine 1, 2, 4, 8, and 12 wk after varicocele induction. Then animals were perfused with phosphate-buffered saline (PBS; Sigma, Germany) to remove blood cells from the testicles. Both the left and right testicles were removed, transferred to liquid nitrogen, and stored for further research at -70 C.

### RNA extraction and real-time polymerase chain reaction (PCR)

Total RNA was extracted from testis tissue in different experimental groups using PeqGOLD RNA TriFast (PeqLab, Germany). A spectrophotometric method at 260/280 nm wavelength was used to determine total RNA amount and quality. 2 μg of total RNA was applied for complementary DNA (cDNA) synthesis in a total volume of 20 μl using RevertAid ${}^{\text{TM}}$ first strand cDNA Synthesis Kit (Aryatous, Iran). Measurement of expression levels of each gene was performed in a 20 μl mixture consisting of 2 μl of cDNA (5-fold diluted), 0.5 μl of 5 mmol/l solutions for each of the forward and reverse primers, and 10 μl of 2x SYBR green DNA PCR Master Mix (Yekta Tajhiz Azma, Iran). Primer sequences are listed in table I. Each sample was duplicated and performed on the LightCyclerⓇ 96 System (Roche, USA). The expression ratio was calculated using a relative formula based on the comparative CT method (2^-ΔΔCt^) (13).

**Table 1:** Primer sets used for amplification


**Genes**		**Primers sequences (5 ' to 3 ' )**	**Product length (bp)**
*Cyclo A*			
	**Sense**	GGCAAATGCTGGACCAAACAC	
	**Antisense**	TTAGAGTTGTCCACAGTCGGAGATG	196 bp
*ASC*			
	**Sense**	GCTGCAGATGGACCCCATAG	
	**Antisense**	ACATTGTGAGCTCCAAGCCA	80 bp
*NLRP3*			
	**Sense**	TCTGTTCATTGGCTGCGGAT	
	**Antisense**	GCCTTTTTCGAACTTGCCGT	314 bp
*Caspase-1*			
	**Sense**	CACGAGACCTGTGCGATCAT	
	**Antisense**	CTTGAGGGAACCACTCGGTC	212 bp
ASC: Apoptosis-associated speck-like protein containing a caspase recruitment domain, NLRP3: Nod-like receptor family pyrin domain containing 3, Cyclo A: Cyclophilin A			

### Ethical considerations

In this study, all experimental protocols were approved by the Ethics Committee of the Arak University of Medical Sciences, Arak, Iran (Code: IR.ARAKMU.RES.1396.234). All ethical protocols for working with laboratory animals were observed in this study.

### Statistical analysis

The results are explained as mean ± standard deviation (SD). Statistical significance was determined using one-way ANOVA or the student *t* test, followed by Tukey's post hoc test in GraphPad Prism 8. P < 0.05 was considered statistically significant.

## 3. Results

**Figure 1:**
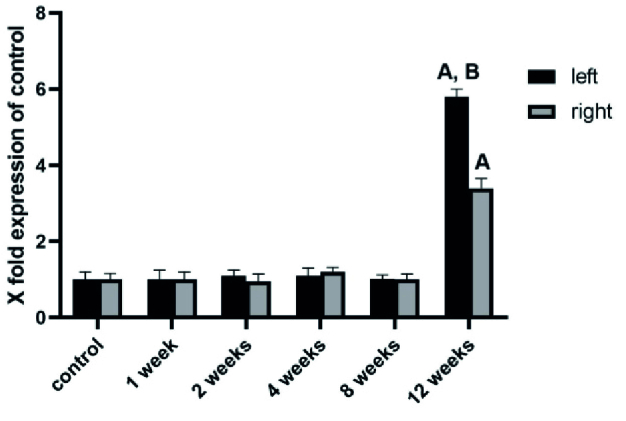
The mRNA expression level of NLRP3 in different experimental groups was analyzed by real-time PCR. Note that during the 1, 2, 4, and 8 wk after varicocele induction, no significant changes were observed in the level of expression of NLRP3 in both testes compared to the control group. 12 wk after surgery, a significant increase was observed in the expression of this gene compared to the control group. A: Significant versus control group, B: Significant versus right testis.

**Figure 2:**
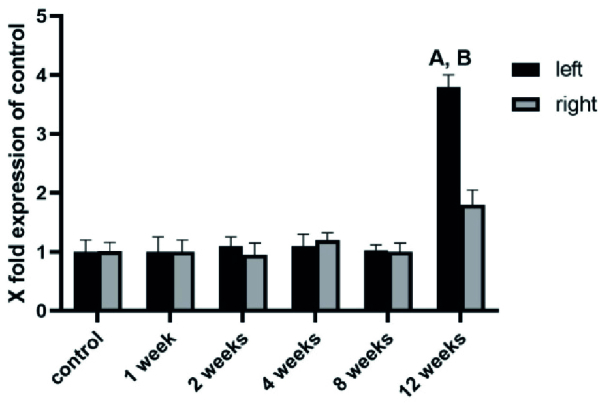
The gene expression level of ASC in all experimental groups. A higher level of ASC expression 12 wk after varicocele induction was shown. A: Significant versus control group, B: Significant versus right testis.

**Figure 3:**
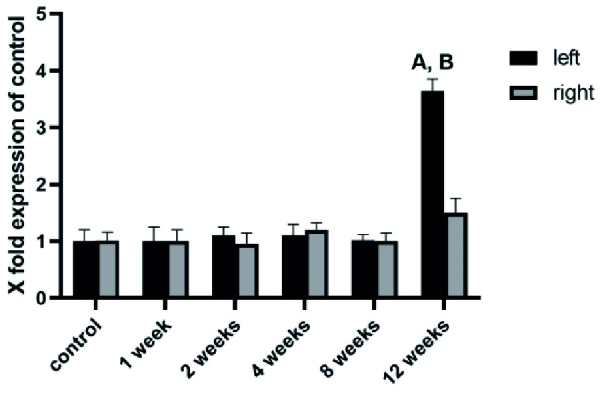
The expression level of caspase-1 in the testis tissue as determined by real-time PCR is presented in different groups. A significant increase was observed in caspase-1 mRNA level 12 wk after varicocele induction, but no changes in caspase-1 expression in the other groups. A: Significant versus control group, B: Significant versus right testis.

We analyzed the mRNA expression of NLRP3, ASC, and caspase-1, the key inflammatory markers, during 1, 2, 4, 8, and 12 wk after varicocele induction in both left and right testes. During the 1, 2, 4, and 8 wk after varicocele induction, no significant changes were observed (p = 0.09) in the level of expression of NLRP3 (Figure 1), ASC (Figure 2), and caspase-1 (Figure 3) in both testes compared to the control group. 12 wk after varicocele induction, the level of expression of the studied genes significantly (p = 0.02) rose in the left testis compared to the control group and the right testis. Interestingly, the mRNA expression of NLRP3 in the right testis was meaningfully increased compared to the control group (p = 0.03) (Figures 1-3).

## 4. Discussion

In this study, we aimed to assess the time course of inflammasome complex expression in testis tissue following varicocele induction. We investigated the expression of NLRP3 inflammasome components in different time points at mRNA levels. As the western blot was not performed, the best time for evaluating the effects of the NLRP3 inflammasome on rat male fertility is 12 wk after varicocele induction.

Although we could not detect any significant changes in the level of expression of the NLRP3 inflammasome before the 12 ${}^{\text{th}}$ wk, changes were reported in NLRP3, caspase-1, and IL-1b mRNA levels 2 months after varicocele induction (16). This difference might be related to the difference in thickness between the wires used for varicocele induction in these 2 studies. In the current study, according to an established protocol, we used a wire of 0.85 mm diameter. It is believed that this thickness is crucial for varicocele induction (17), but by using a thinner wire (0.64 mm) the results will be different (16).

According to the previous study, proinflammatory cytokines such as IL-1α and IL-1b increased 11 and 13 wk after varicocele induction, respectively (18).

It seems that, concerning the type of tissue and severity of the injury, a difference was observed in the expression pattern of the inflammasome complex. In some pathological conditions such as SCI (13) and stroke (19), in which ischemia and neuronal damage lead to acute and chronic responses in the neuronal tissue, the inflammasome complex rises during the first 72 hr after injury. Given that in our study, 12 wk after varicocele induction, we could detect changes in the NLRP3 inflammasome expression level, this long time for altering the level of expression of the inflammasome complex might be related to the pathogenesis of varicocele. Due to the venous blood retention and increasing hydrostatic pressure that occurs in the testis following varicocele, these factors cause hypoxia in the testicular tissue (20); therefore, it takes a long time for the inflammatory factors in the testis to increase. Hypoxia and its related signaling pathway are believed to be responsible for varicocele pathogenesis (21). Previous studies reported that by targeting the inflammasome complex activity in some pathological conditions, therapeutic strategies would be developed (22, 23).

In our previous work, we used omega-3 polyunsaturated fatty acids immediately after stroke induction to downregulate the inflammasome activity and reduce the side effects of stroke on brain tissue (24). NLRP3 inflammasome activation was inhibited by BAY 11-7082 and A438079 in a mouse model of SCI to improve neurological recovery (25). It is suggested that using MCC950, a small molecule for deactivating the NLRP3 complex, reduces atherosclerotic plaque formation and improves vascular function in diabetic mice (26). Given that inhibiting the inflammasome complex reduces the destructive effects of varicocele on testis tissue (15), controlling the expression of the NLRP3 inflammasome as adjuvant therapy appears to be a promising target for alleviating varicocele complications (16).

## 5. Conclusion

Our results determined that 12 wk after varicocele induction is the best time for evaluating the role of the NLRP3 inflammasome in the pathogenesis of varicocele. Finding this time course of inflammasome activity is important for clarifying the mechanism related to the NLRP3 complex in varicocele for further studies.

##  Conflict of Interest

The authors declare that there is no conflict of interest.
